# Green chemistry routed sugar press mud for (2D) ZnO nanostructure fabrication, mineral fortification, and climate-resilient wheat crop productivity

**DOI:** 10.1038/s41598-024-53682-0

**Published:** 2024-02-19

**Authors:** Lahur Mani Verma, Ajay Kumar, Ashwani Kumar, Garima Singh, Umesh Singh, Shivani Chaudhary, Sachin Kumar, Anita Raj Sanwaria, Pravin P. Ingole, Satyawati Sharma

**Affiliations:** 1 Biomass Technology Laboratory, Centre for Rural Development and Technology (CRDT), Indian Institute of Technology Delhi (IITD), Room No. 289, Block-III, Main Building Hauz Khas, New Delhi, 110016 India; 2 Metagenomics and Secretomics Research Laboratory, Department of Botany, University of Allahabad (A Central University), Prayagraj, 211002 UP India; 3 Biommaterials and Bio-Interface Laboratory, Center for Biomedical Engineering IIT Delhi, New Delhi, 110016 India; 4 Electrophysical Laboratory, Department of Chemistry, IIT Delhi, New Delhi, 110016 India

**Keywords:** Biogeochemistry, Environmental sciences, Chemistry, Engineering, Materials science

## Abstract

Nanotechnology appears to be a promising tool to redefine crop nutrition in the coming decades. However, the crucial interactions of nanomaterials with abiotic components of the environment like soil organic matter (SOM) and carbon‒sequestration may hold the key to sustainable crop nutrition, fortification, and climate change. Here, we investigated the use of sugar press mud (PM) mediated ZnO nanosynthesis for soil amendment and nutrient mobilisation under moderately alkaline conditions. The positively charged (+ 7.61 mv) ZnO sheet-like nanoparticles (~ 17 nm) from zinc sulphate at the optimum dose of (75 mg/kg blended with PM (1.4% w/w) were used in reinforcing the soil matrix for wheat growth. The results demonstrated improved agronomic parameters with (~ 24%) and (~ 19%) relative increases in yield and plant Zn content. Also, the soil solution phase interactions of the ZnO nanoparticles with the PM-induced soil colloidal carbon (− 27.9 mv and diameter 0.4864 μm) along with its other components have influenced the soil nutrient dynamics and mineral ecology at large. Interestingly, one such interaction seems to have reversed the known Zn-P interaction from negative to positive. Thus, the study offers a fresh insight into the possible correlations between nutrient interactions and soil carbon sequestration for climate-resilient crop productivity.

## Introduction

Recently, nanomaterials especially the metal oxides (M_*x*_O_*y*_) have entered agriculture as a promising material for fighting the low nutrient use efficiency (NUE) associated generally with bulk-regime conventional agrochemicals^1^. However, the several aspects of the nanomaterial environment interface are either poorly understood or the information available is insufficient against the vast variance in geography-based agro-climatic conditions^2,3^. Further, this variation in the biotic (soil microbial flora, rhizospheres) and abiotic (soil organic matter, mineral matrix, soil pH, etc.) components of the environment over space and time makes the design, and production of the suitable nanomaterial a big challenge^3–5^. The reported use of nanomaterial like ZnO NPs as fertilizer is either incomplete in its characterisation or poorly described. Also, there is a lack of information on their behaviour in environment, especially their crucial interactions with biotic and abiotic components of the environment^2,6^. Additionally, the sustainable production of nanomaterials for their projected vast-scale application in agriculture is also a challenge. While the chemical synthesis of the nanomaterial like ZnO, NPs is generally seen as unsustainable, the greener approaches are often associated with poor synthetic protocols and over-reliance on natural vegetation^7^. Further, the consistent synthetic outcome of greener approaches is largely unaddressed. In our recent report, we have shown some levels of preliminary consistency in the synthetic outcome of a sugar press mud (agro-waste) led two-fold green synthesis of ZnO nanoparticles. This has consistently yielded a sheet-like wurtzite, P63*mc* nanocrystals with the zinc sulphate (ZnSO_4_.7H_2_O) as precursor^7^. Thus, this has shown that a waste-led process cannot just reduce or even replace the over-reliance on green vegetation but also seems to have a potential for the synthetic outcome-controlled sustainable process for ZnO NPs production^7^. Moreover, the behaviour of nanomaterials like ZnO, NPs strongly depends on their physical and chemical properties. But a very few reports have attempted this correlation in structure and activity with respect to the environment interface^8^. However, few recent reports have shown that the nanomaterials like, metal oxides (M_x_O_y_), nanocomposites, and nanoemulsions including ZnO NPs in different shapes (rod, sphere, needle etc.) and sizes (20–150 nm), can offer a median gain nutrient use efficiency (NUE) over their bulk analogues or the conventional fertilizers^1,9–11^. But these results are far from presenting a complete picture in terms of mechanisms underlying these gains in efficiency, especially their multifaceted interactions with various components of the environment^4,5^. Notably, few recent reports have emerged highlighting the utilisation of one such interface i.e. mineral-soil organic matter (SOM) interaction in achieving the required nutrient use efficiency (NUE) of the fertilisers. This might be helpful in improving the use efficiency (NUE) of nutrients especially for the zinc-based fertilisers, which is very low (2–5%) for the current zinc salt based conventional fertilizers^3,12–14^. This approach of utilising the ZnO –SOM interaction interface may offer the required advantage of nutrient use efficiency due to its solubility-driven slow release moderated further by carbon sequestration. Thus, collectively leading to sustainable agriculture for a climate-resilient future^15–17^.

This report presents a fresh perspective on sugar industry-based solid waste (PM) led green synthesis of ZnO nanoparticles using an aqueous sol–gel process. This is followed by its application toward the added utilisation of PM in increasing the soil organic carbon (SOC) and nutrient mobilisation for the mineral fortification and nutrient use efficacy of ZnO NPs in a soil mode application experiment. This might be an appropriate and circular approach in meeting the increasing demand for global food production and fighting the widespread hidden hunger and environmental problems.

## Materials and method

### Procurement of reagents and other materials

All the chemicals used in the experiments were of analytical grade and were obtained from Sigma Chemicals (USA). Sugar press mud (PM) was procured from TR Solvents Pvt. Ltd. (Faridabad, Haryana, India). Wheat (*Triticum aestivum L.*) seeds HD-3226 were obtained from the Indian Council of Agricultural Research—Indian Agricultural Research Institute (ICAR—IARI), Pusa New Delhi, in accordance with the national and international guidelines.

### Sugar press mud analysis

The characterisation of sugar press mud was done using various techniques. The chemical functional groups associated with dry PM were analysed in the transmittance mode of an FTIR spectrophotometer (Perkin-Elmer1600). The colloidal suspension of PM in water was analysed using (DLS) Zetasizer Ver. 7.11 (Malvern Instruments). The mineral profiling of dry sugar press mud was done (ICP-MS, Agilent Technologies make Model: 7900) following acid-based microwave digestion. The mineral mapping used SEM-EDX (Oxford-EDX system IE 250 X Max 80, Netherlands).

### Synthesis and characterisation of ZnO NPs

Zinc oxide (ZnO) NPs were prepared using the sol–gel method following the procedure described in our previous report by Verma et al.^7^. The dried sample was characterised using techniques viz. X-ray diffraction; Instrument: XRD X’Pert Pro (PANalytical Netherlands), microscopy Instruments: SEM–EDX (Oxford-EDX system IE 250 X Max 80, Netherlands) and TEM (JEOL JEM-1400), FTIR spectrophotometer (Perkin-Elmer1600) in transmittance mode and UV–Vis absorption Epoch 2 microplate reader (Biotek instruments). The Raman spectrum was recorded using FT-IR Raman spectrometer with microscope—Varian 7000; Instrument: FT-Raman and Varian 600 UMA. DLS Zetasizer Ver. 7.11 (Malvern Instruments) was used for measuring the hydrodynamic diameter and zeta (ζ) potential^18^. The summary of synthesis conditions is given in Table S1.

### Soil analysis

The soil used in this study was collected from micromodel complex (CRDT, IIT Delhi, New Delhi, India coordinates (28° 32′ 42″ N, 77° 11′ 32″ E). The soil sampling and analysis were done following the protocol described by Yusefi-Tanha et al.^11^. The physicochemical analysis of soil samples like electrical conductivity (EC) and the pH estimation was done using a pH meter (pH510 Eutech and EC meter CON 510 Eutech instruments, India) by preparing soil: water suspension 1:10 (pH) and 1:5 (EC) respectively^19^. The CHN analyser (CHNOS Elementary, Vario EL III model) was used for soil samples' C, H, and N analysis. For mineral analysis (ICP-MS, Agilent Technologies make Model: 7900), microwave acid digestion was used. The post-harvest analysis of soil samples was done for minerals, CHN and exchangeable Zn following the protocol described above.

### Experimental setup

The completely randomised control pot trial (Fig. S1) experiment was conducted between November and May 2020–21 at micromodel complex, CRDT IIT Delhi, New Delhi, (India) under the regional agro-climatic conditions (temperature 8–40 °C, average relative humidity 32–45%) in an area enclosed by a garden net which allows approximately 95% sunlight and air. This randomised control block design (RCBD), as shown in (Fig. S1), was set up following the procedure described^20^. The details of the treatment design are described in Table 1. This experimental setup (prepared treatments) was left to equilibrate with the outside environment overnight and followed by sowing of the surface sterilised (2% H_2_O_2_) seeds (10 grain per pot) of the wheat cultivar (HD-3226, at 5 cm depth in the pot (x = 10) plants. The seeds were subsurfaced in the soil manually. No other inputs (other than those described in the treatments) were added before or after the experiment's setup. The experiment was monitored during the complete life cycle of wheat.

**Table 1:** Detailed treatments.

Sr. No.	Symbol	Treatment details
1	T0	Control (3 kg sandy loam soil)
2	T1	Soil and Nano zinc oxide (ZnO) 75 mg/kg
3	T2	Soil and ZnSO_4_.7H_2_O Agricultural grade 75 mg/kg
4	T3	Soil, sugar press mud (1:4 w/w) and Nano ZnO 75 mg/kg
5	T4	Soil and sugar press mud (1.4% w/w)

### Measurements of chlorophyll and plant growth parameters

The photosynthetic pigments (chlorophyll ‘a’ and ‘b’) were estimated for all treatments at their boot stage (145 days), following random sampling of leaves from three replicates of the different treatments. The sample preparation and chlorophyll measurement were done following the detailed protocol by Ozdemir et al.^21^. The amount of chlorophyll content was calculated using the following Eq. (1).

1 $$\begin{gathered} {\text{Chl}}\;{\text{a}}\left( {{\text{mg}}/{\text{ml}}} \right) = {11}.{64} \times \left( {{\text{A663}}} \right) - {2}.{16} \times \left( {{\text{A645}}} \right) \hfill \\ {\text{Chl}}\;{\text{b}}\left( {{\text{mg}}/{\text{ml}}} \right) = {2}0.{96} \times \, \left( {{\text{A645}}} \right) - {2}.{16} \times \, \left( {{\text{A663}}} \right) \hfill \\ \end{gathered}$$

For growth parameters, the plant samples of different treatments were collected after harvesting the crop, and their growth (phenotypic) parameters, viz. biomass, plant height and fruit weight, were analysed following the detailed protocol by Singh et al.^22^, with slight modifications. In short, the harvested wheat plants were air-dried to a constant weight and weighed after being separated into the shoot (stem, leaves), panicle, and grain. For elemental analysis (Zn, Cu, Mg, and Fe), sampled plant parts were firstly converted into coarsely powdered form, followed by acid digestion following a slightly modified protocol described by Tüzen^23^, and analysed using the ICP-MS technique with similar protocol as above.

### Statistical analysis

The measurements of the triplicate values were recorded and expressed as Mean ± Standard deviation (SD). The statistical analysis with SPSS software (version 21.0) was performed using a one-way analysis of variance (ANOVA). The mean separations were performed using Duncan’s multiple range test (DMRT), with *p* < 0.05 considered significant. PCA analysis was used to envision the experimental values that explained the co-relationship pattern between the set of observed variables, which was analysed using Minitab version (21.1.0.0).

## Results and discussion

### Characterisation of sugar press mud

**Figure 1:**
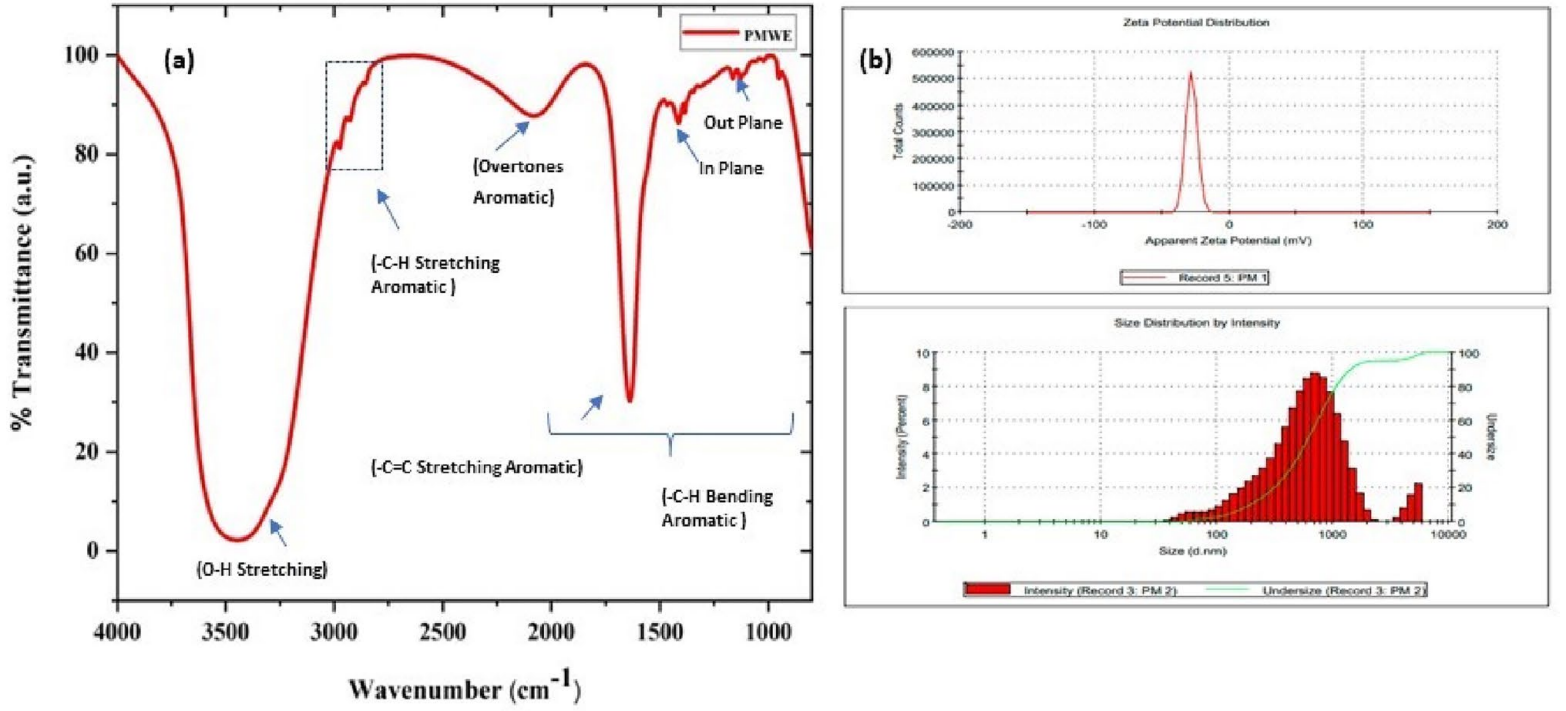
(**a**) FTIR shows the associated functional groups with dry PM, and (**b**) shows the size and charge of colloidal particles of PM in water.

**Figure 2:**
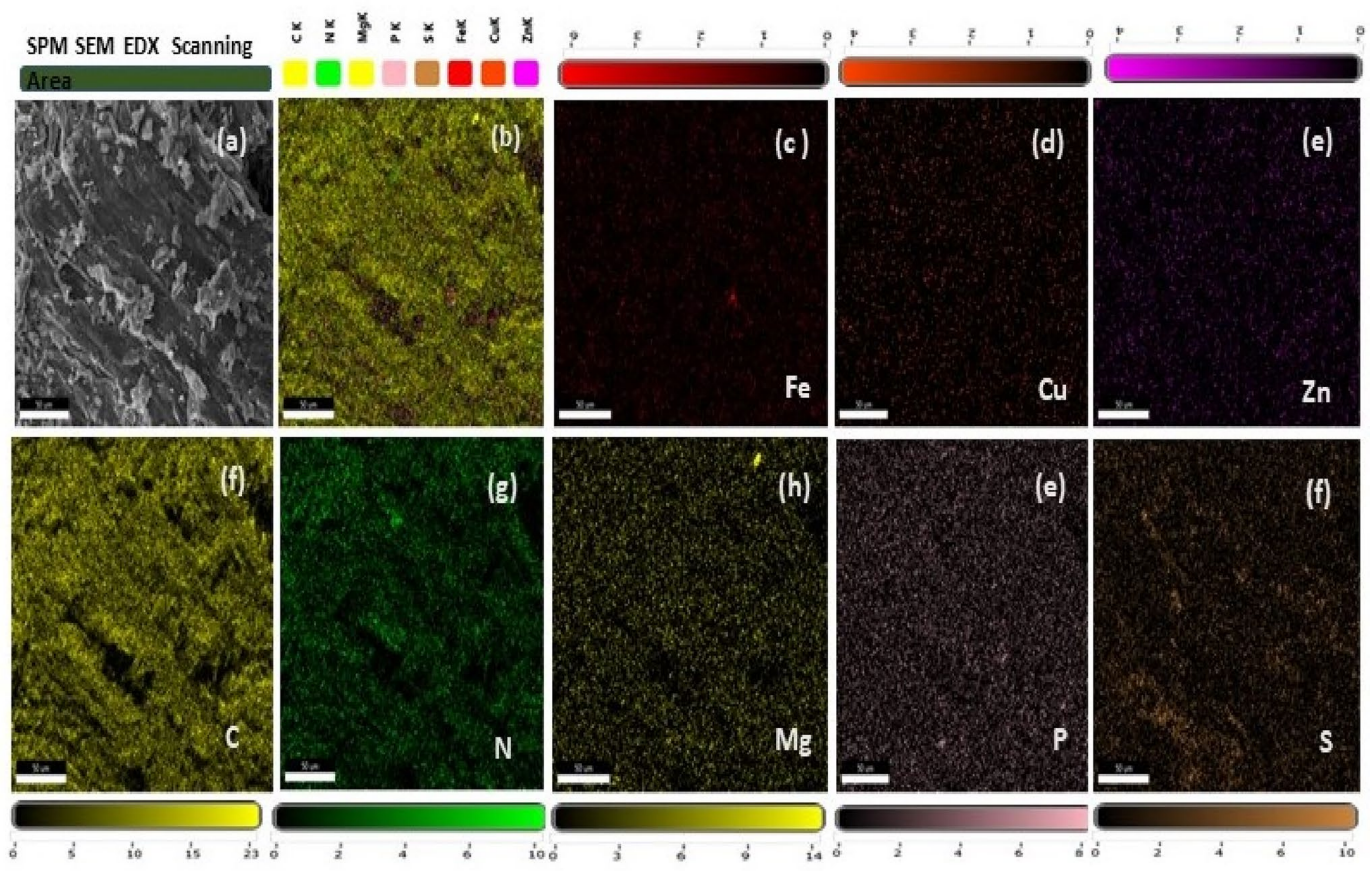
SEM/EDX (**a**–**f**) mapping analysis of dry sugar press mud sample for its mineral composition. The EDX images (**b**–**f**) show poor mineral presence in samples.

The dry press mud was characterised for its texture, chemical functionalities, mineral profile, and colloidal properties using SEM, FTIR, EDX, ICP-MS and DLS techniques. The press mud (PM) characterisation is generally limited to the proximate analysis of its physicochemical properties and chemical composition^24^. According to the previous studies, the (PM) chiefly contains cellulose, hemicellulose, and lignin as a fibre, adding up to 15–30% along with 5–15% sugar of its total constituent. Beyond this, it also contains 5–14% crude wax and crude protein up to 5–15% as organic components with some free functional groups like aldehyde, ketone, lactone, and amines. It also contains some aromatic and aliphatic hydrocarbons, vis-a-vis inorganic salts, as indicated in FTIR spectra Fig. 1(i)^24^. Moreover, a study by Velmurugan^25^ reported the recovery of some useful chemicals from extracts of press mud (PM) in a pH-dependent manner. This extract was constituted with 0.85% sugar and 3.3% protein. The dry press mud analysis using ICP-MS (Table S2) and SEM/EDX mapping (Fig. 2, and Table 2) have provided its mineral profiling, which shows that PM is rich in carbon (~ 70%) and nitrogen (~ 20%), with a moderate level of phosphorous and sulfur (~ 2%). However, it has low status of essential minerals (Table S2) like Cu, Fe, and Zn (0.1–0.2%). Further, the colloidal suspension of dry PM in water shows particle surface charge and diameter (DLS) of the order of − 27.9 mv and 0.4864 μm, respectively (Fig. 1(ii)). The probable chemical agents present in PM water extract acting as capping agent in nanosynthesis were analysed in our previous report by Verma et al.^7^. This chemical profile makes it suitable for nanosynthesis and the study of soil carbon sequestration and mineral-organic interface.

**Table 2:** Summary of elemental composition of PM (SEM/EDX) analysis.

Element	C	N	Mg	P	S	Fe	Cu	Zn
Weight (%)	73.7	22.2	0.2	1.8	1.9	0.1	0.1	0.2
Atomic (%)	78.2	20.2	0.1	0.7	0.8	0	0	0
Error (%)	8.6	11.7	8.5	2.7	3.2	62.8	69.4	8.1

### Characterisation of ZnO nanoparticles

**Figure 3:**
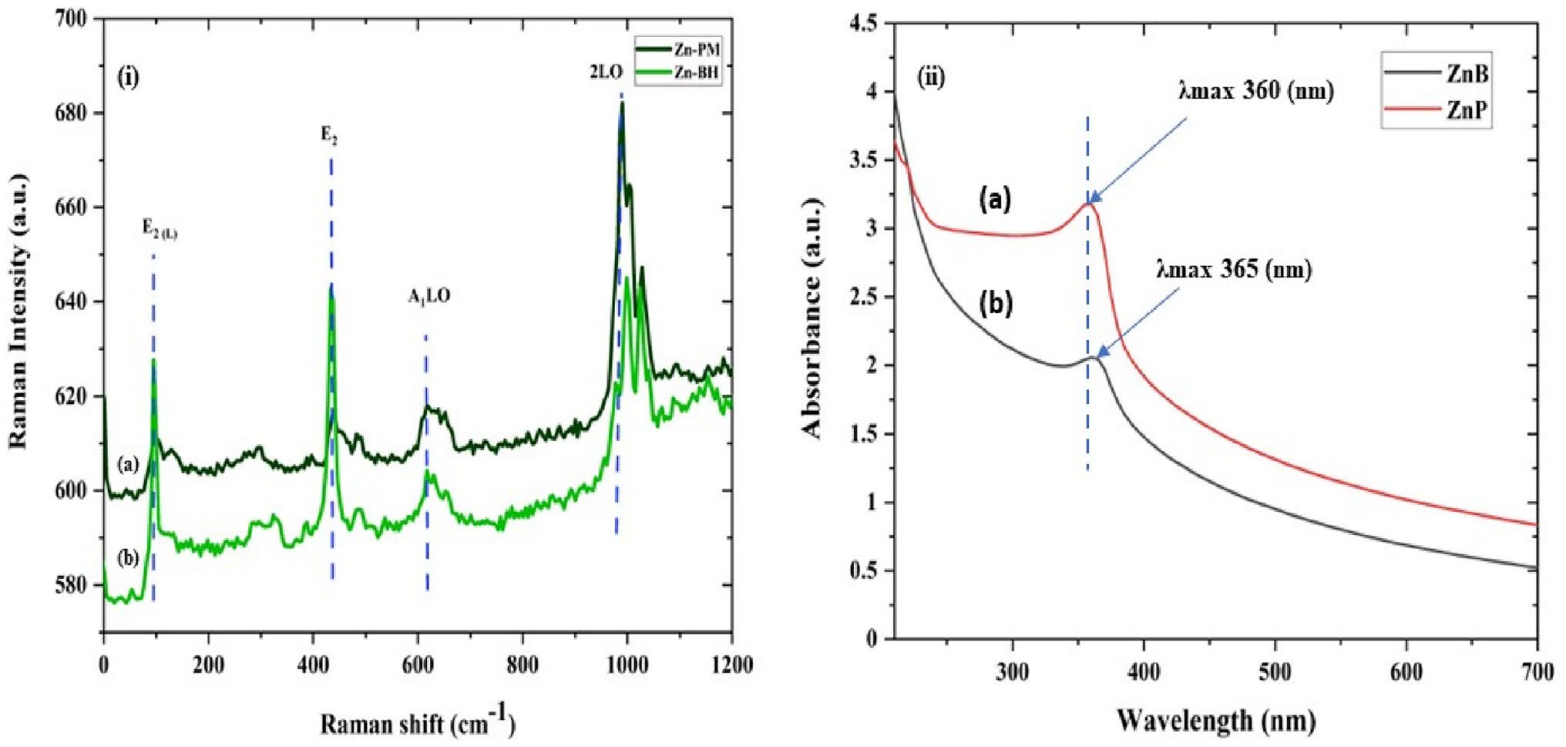
(i) Raman spectra of ZnO NPs; with characteristic Raman shifts (ii) UV- absorption spectroscopy of ZnO NPs showing blue shift in absorption maxima (λmax values, i.e. 360–365 nm indicating quantum confinement in nanomaterial due to reduction in dimension (15–25 nm).

**Figure 4:**
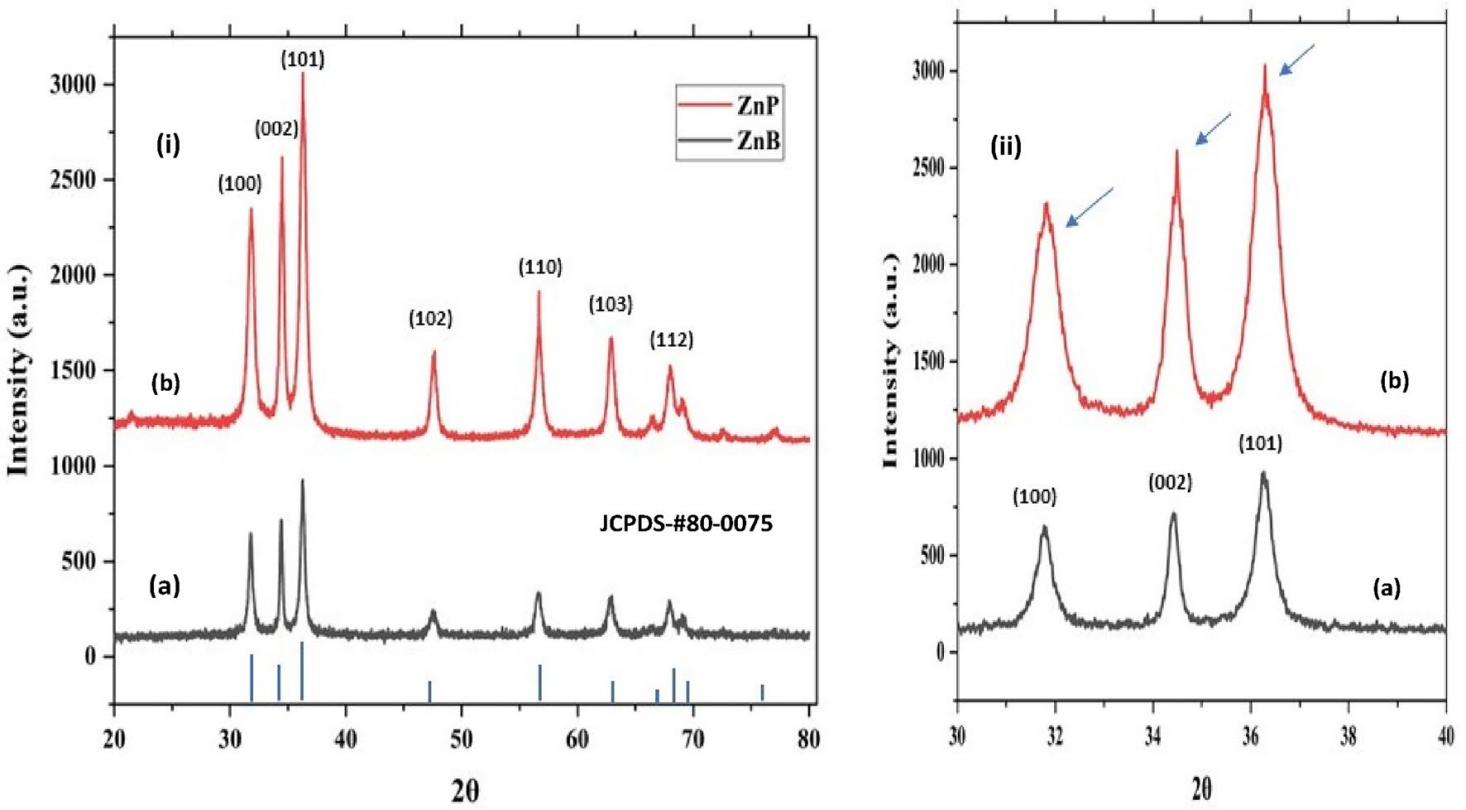
(i) X-ray characteristic diffractograms for ZnO nanoparticles, JCPDS no. (80–0075); the relative intensities of the peaks (100), (002), (101) magnified (ii) are indicative of the preferred orientation due to 2D morphology (b) compared to ZnB (a).

**Figure 5:**
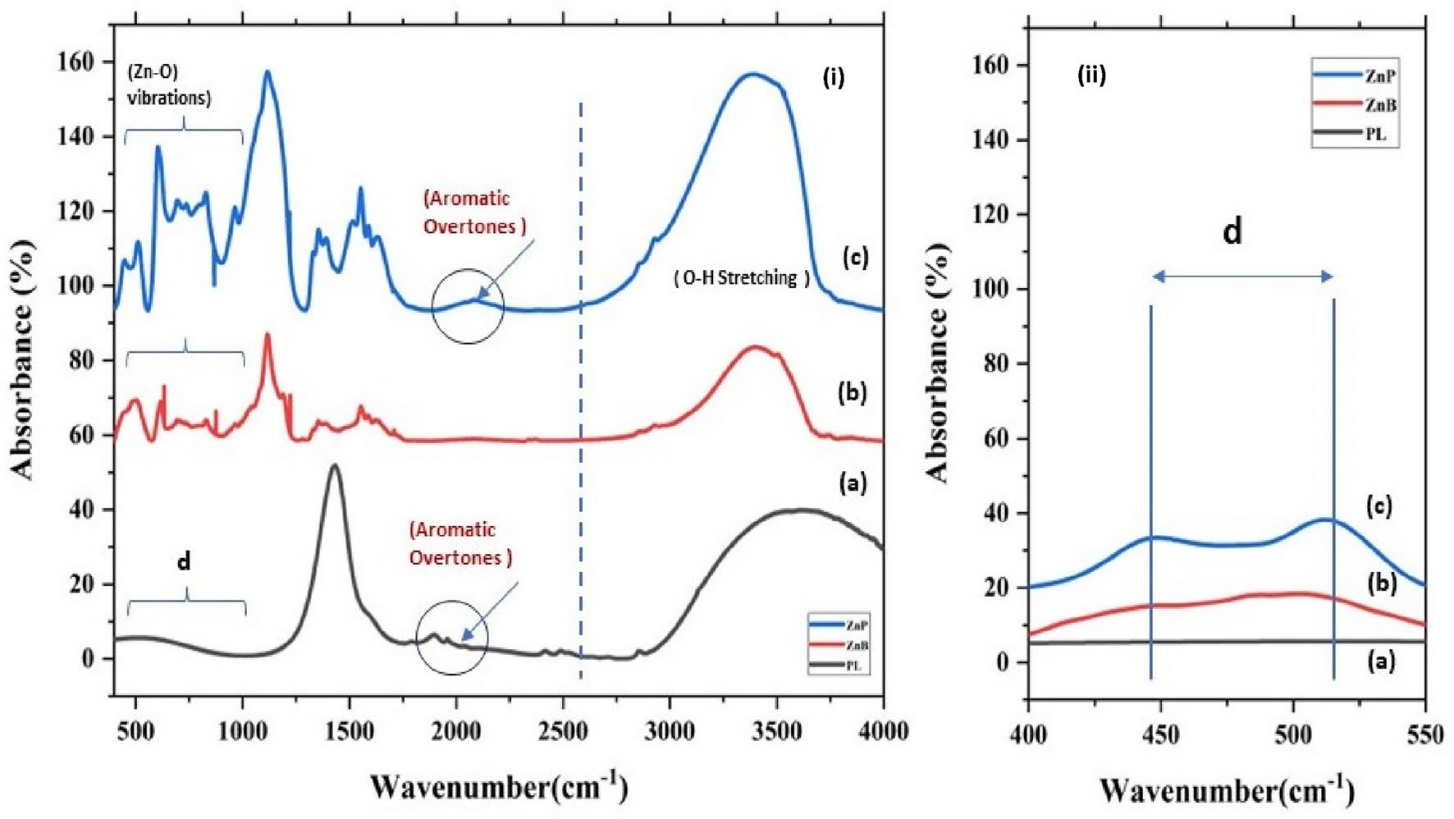
FTIR spectra for characteristic Zn–O stretching, the splitting of the characteristic speak (i&ii) c) (400–550 cm^−1^) is indicative of different axial ratio (a/b > 1) due to 2D sheet-like morphology; the residual overtone peak indicates the traces of capping ligand on the particle surface.

**Figure 6:**
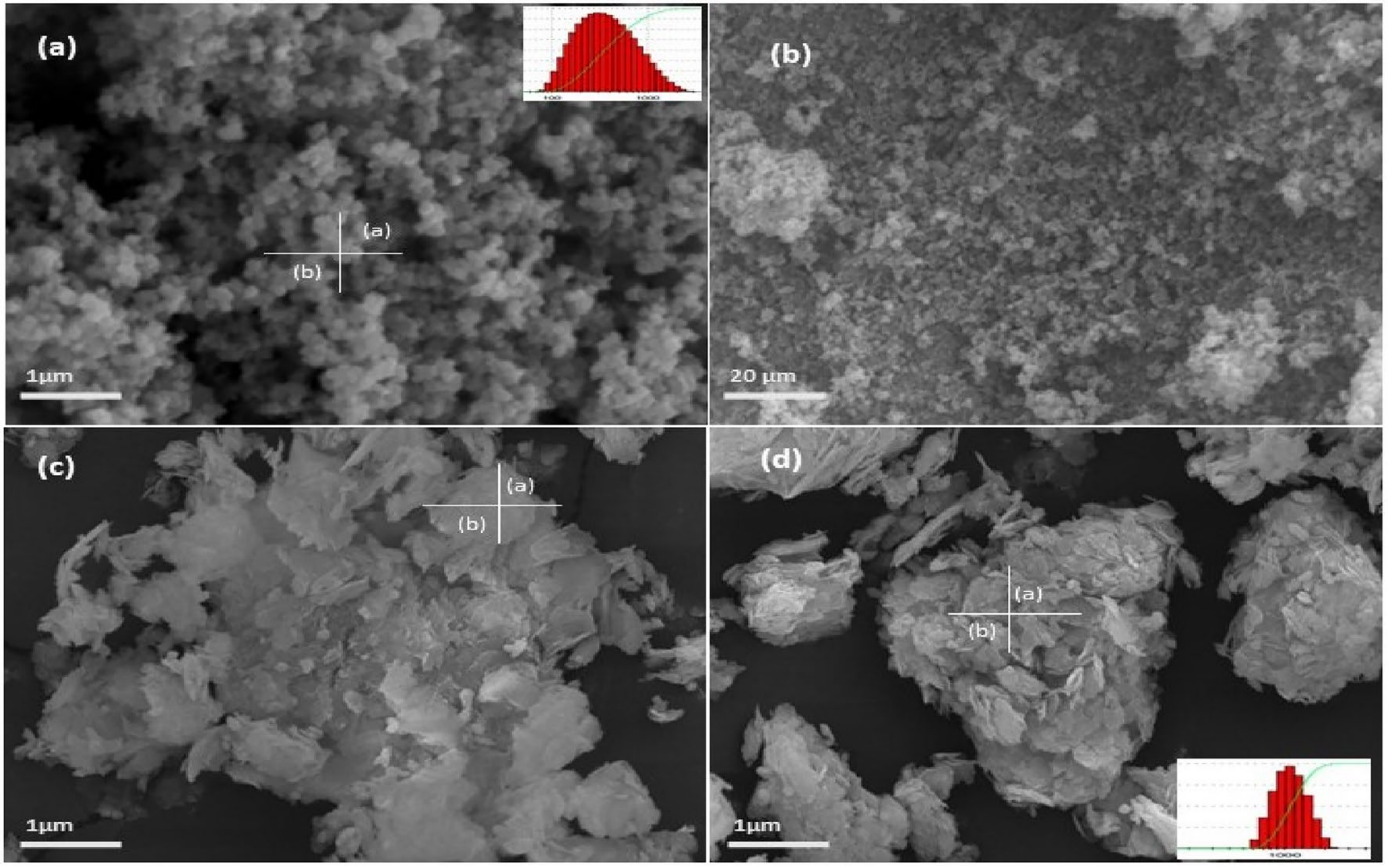
SEM images (**a**–**d**) showing sphere and sheet-like morphologies of ZnO NPs based on the aspects ratio of the nanoparticles (a/b ≥ 1) in two cases ZnB (**a**, **b**) and ZnP (**c**, **d**) indicate the changes in the morphology due to the role played by the green ligand. The inset a(i) and d(i) shows the polydispersity of the material.

**Figure 7:**
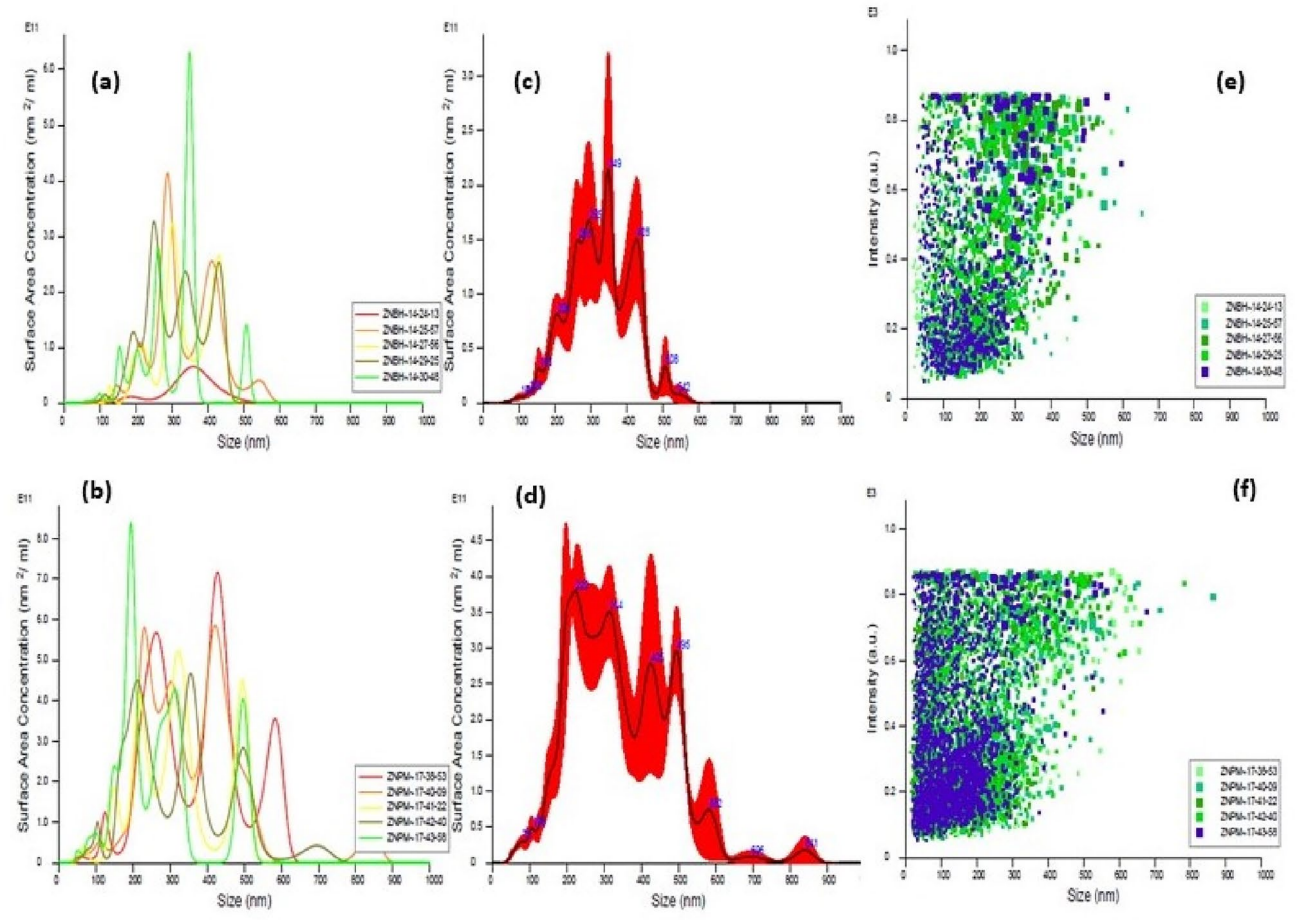
The modal size distribution of the ZnO nanoparticles, i.e. 221 nm in ZnP (**b**–**f**) and 348 nm in ZnB (**a**–**e**) using Nanoparticle Tracking Analysis (NTA **a**–**f**) indicates the different available surface area to ZnO nanoparticles in the units of m^2^/ml.

**Figure 8:**
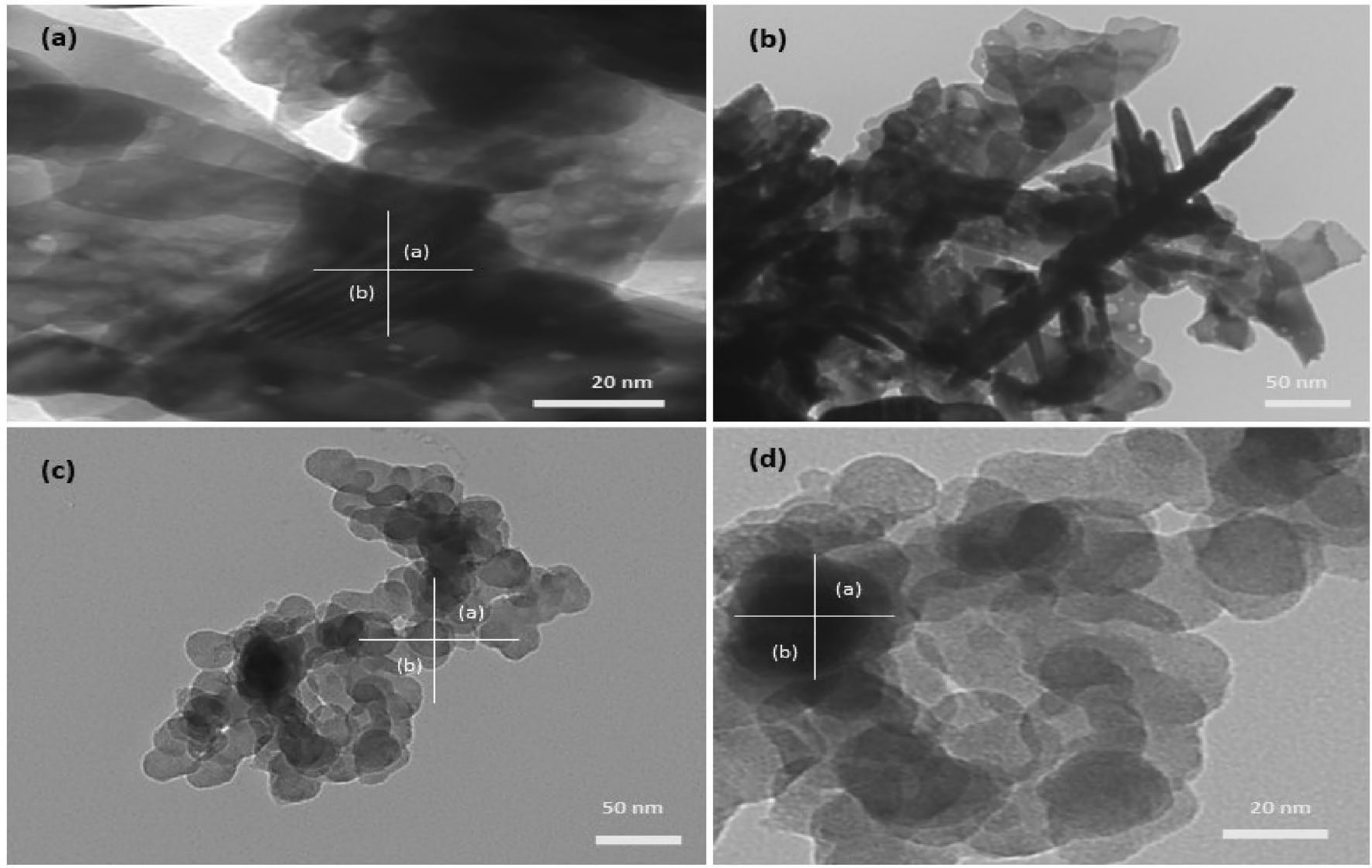
TEM images (**a**–**d**) confirm the sphere and sheet-like morphologies ZnO NPs based on the aspects ratio of the nanoparticles (a/b ≥ 1) in two cases; sphere-like ZnB (**a**–**b**), and sheet-like ZnP (**c**–**d**) indicate the changes in the morphology due to the role played by the green ligand.

The ZnO NPs were synthesised (summary, Table S1) using the sol–gel method (pH = 12) and ambient reaction temperature (~ 35 °C). The X-ray diffraction analysis of sintered (vacuum 85 °C) powder using Debye Scherrer Equation

2 $$\left(\tau \right)= \frac{k (\lambda )}{\beta (\mathit{cos}\theta )}$$

(where $\left(\tau \right)$ is average crystallite size, λ is the wavelength for Cu Kα radiation 1.5406 A˚, β is the full width at half maximum (radian) taken corresponding to (101) plane, and (θ) is the brag angle in degrees) along with Raman spectra (Fig. 3i) which confirms the wurtzite (P63*mc*) phase (JCPDS-#80–0075) of ZnO nanoparticles. The average crystallite size calculated using this Eq. (2) was found to be in the order of ~ 17 (ZnP) to ~ 26 (ZnB) nm, respectively. This is also indicated by the two samples' quantum confinement-driven blue shift (λmax 360–365 nm) in the UV visible absorption spectrum (Fig. 3ii). Further, the apparent relative intensity ratio of the three XRD peaks corresponding to miller indices h k l (100), (002) and (101) (Fig. 4i–ii) due to the preferred face orientation of nanocrystals suggests the non-spherical (aspect ratio a/b where a ≠ b) nanostructures in the case of ZnP sample^26–28^. This underlines the role of some capping agents present in the PM extract^7^. This observation is supported further by the fact that the characteristic peaks (ZnP) of Zn–O stretching around frequency (ν) 400–550 nm in the FTIR spectrum (Fig. 5i–ii) appear splitted and asymmetric, with respect to the ZnB sample. This indicates the non-spherical shape (ZnP) due to the altered axial ratio of the vibrating dipole, according to the theory of average dielectric constant (TDAC)^27^. Further, the polydispersity index (PDI) values 0.325 (ZnP) and 0.533 (ZnB) (table S1) in dynamic light scattering (DLS) studies Fig. 6a(i) & d(ii) and reversal of zeta potential (ζ) − 2.27mv ZnB and + 7.61mv (Table S1) shows the narrower size distribution, and higher colloidal stability of nanoparticles in the case of ZnP—suggesting the effective role played by the ligands present in the PM extract^29^. This is also reflected in the nanoparticle tracking (NTA) analysis (Fig. 7(a–f)) as an apparent increase in total available surface area in units of nm^2^/ml of the sample ZnP compared to ZnP as evident by the difference in modal distribution of different sized nanoparticle in both the cases. The SEM (Fig. 6(a–d)) and TEM (Fig. 8(a–d)) images of the samples confirm all these observations, where the difference in the morphology, i.e. sheet-like in the case of ZnP (with capping agent) and ZnB (without capping agents) is evidenced by the fact of clearly different aspect ratio (a/b ≥ 1) of the nanoparticles. All these observations, such as the significant variation in morphology, charge, and dispersity but less pronounced variation in size, highlight the specific behaviour of the capping agent present in the water extract of PM, which has been explored in some detail by Verma et al.^7^ and needs further exploration.

### Soil analysis

The soil used in the experiment was analysed for its various physico-chemical parameters. The soil morphology is sandy loam with low soil carbon status and slightly alkaline pH. The detailed parameters of the soil are given in Table 3. Briefly, the soil used in the pot experiments was found to be slightly alkaline (pH = 8.72 ± 0.10), with a sandy loam texture, which is suitable for controlled study of mineral-soil organic carbon interface primarily due to the low soil organic (SOC) level and sandy loam texture. The soil has shown solvated ion-based electrical conductivity (EC, mS cm^−1^) and percentage (% w/w) SOC content at 0.30 ± 0.04 and 0.72 ± 0.03, respectively. The NPK content of soil (in mg kg^−1^) was found to be 135.00 ± 12.30, 349.12 ± 0.30, and 3461.30 ± 8.50, respectively.

**Table 3:** Structural and physio-chemical characterisation of soil used in the experimental study.

S.No	Soil property	Mean value ± SD
1	pH w (1:2.5; w/v)	8.72 ± 0.1
2	EC (mS cm^−1^)	0.3 ± 0.04
3	Soil Organic Carbon (SOC) (% w/w)	0.72 ± 0.03
4	N (mg kg^−1^)	135 ± 12.3
5	P (mg kg^−1^)	349.12 ± 0.3
6	K (mg kg^−1^)	3461.30 ± 8.5
7	Total Zn (mg kg^−1^)	143.0 ± 4.2
8	DTPA-extractable Zn (mg kg − 1)	1.4 ± 0.1
9	Cd (mg kg^−1^)	0.93 ± 0.02
10	Mg (mg kg^−1^)	2565.34 ± 5.1
11	Sand (%w/w)	70.8 ± 2.0
12	Silt (%w/w)	15.2 ± 1.0
13	Clay (% w/w)	14.1 ± 1.2
14	Texture (USDA)	Sandy loam

EC, Electrical conductivity; DTA, Diethylenetriaminepentaacetic Acid, SOC, Soil Organic Carbon.

### Chlorophyll content, plant growth and yield

**Figure 9:**
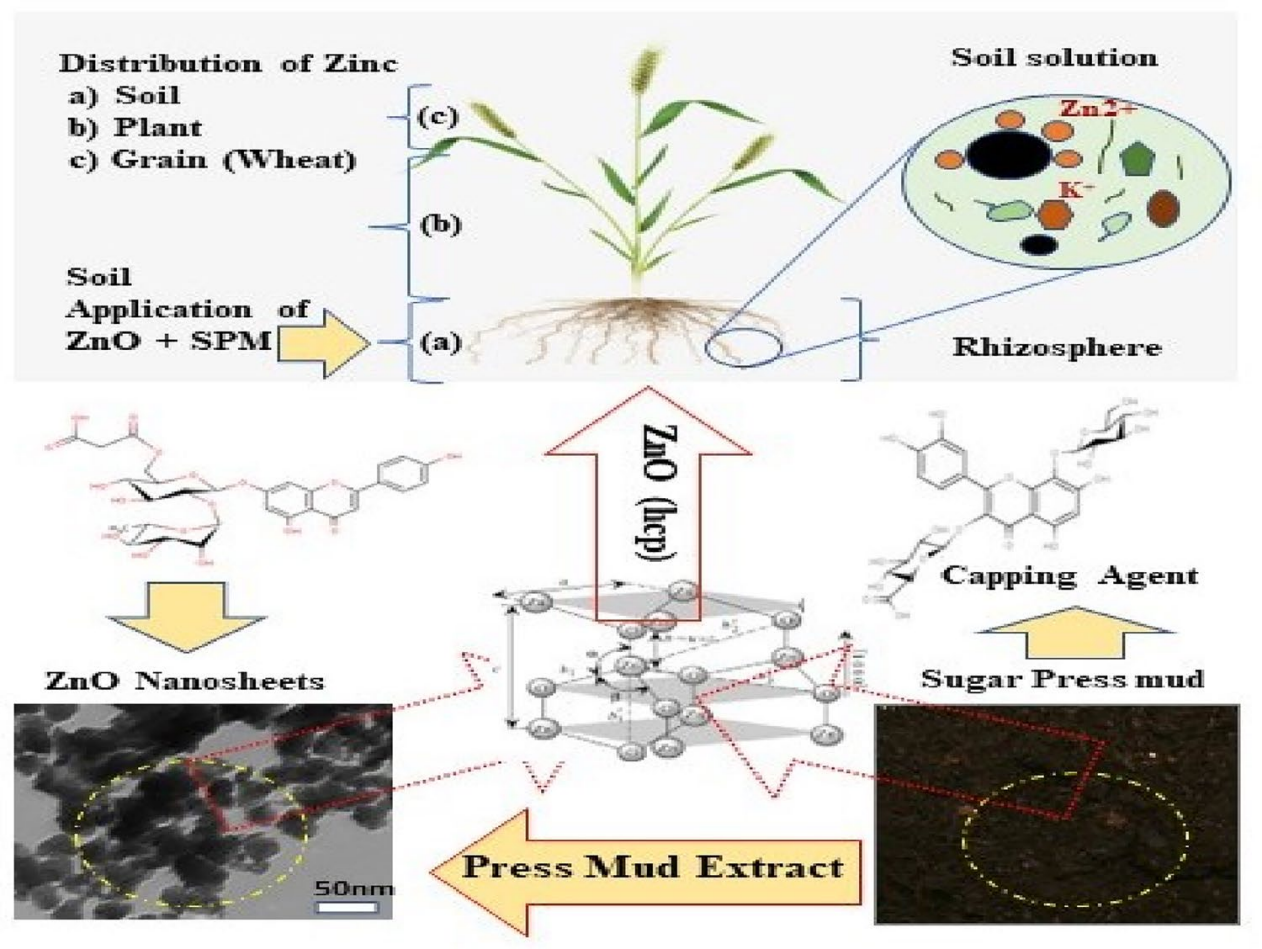
A schematic diagram showing the whole process from the synthesis of the ZnO NPs to its application; The interaction between negatively charged soil colloidal carbon surface with positively charged ZnO NPs (sheet-like structure); nano surfaces along with other mineral nutrients interactions in soil solution for nutrient uptake efficiency has also been shown.

**Figure 10:**
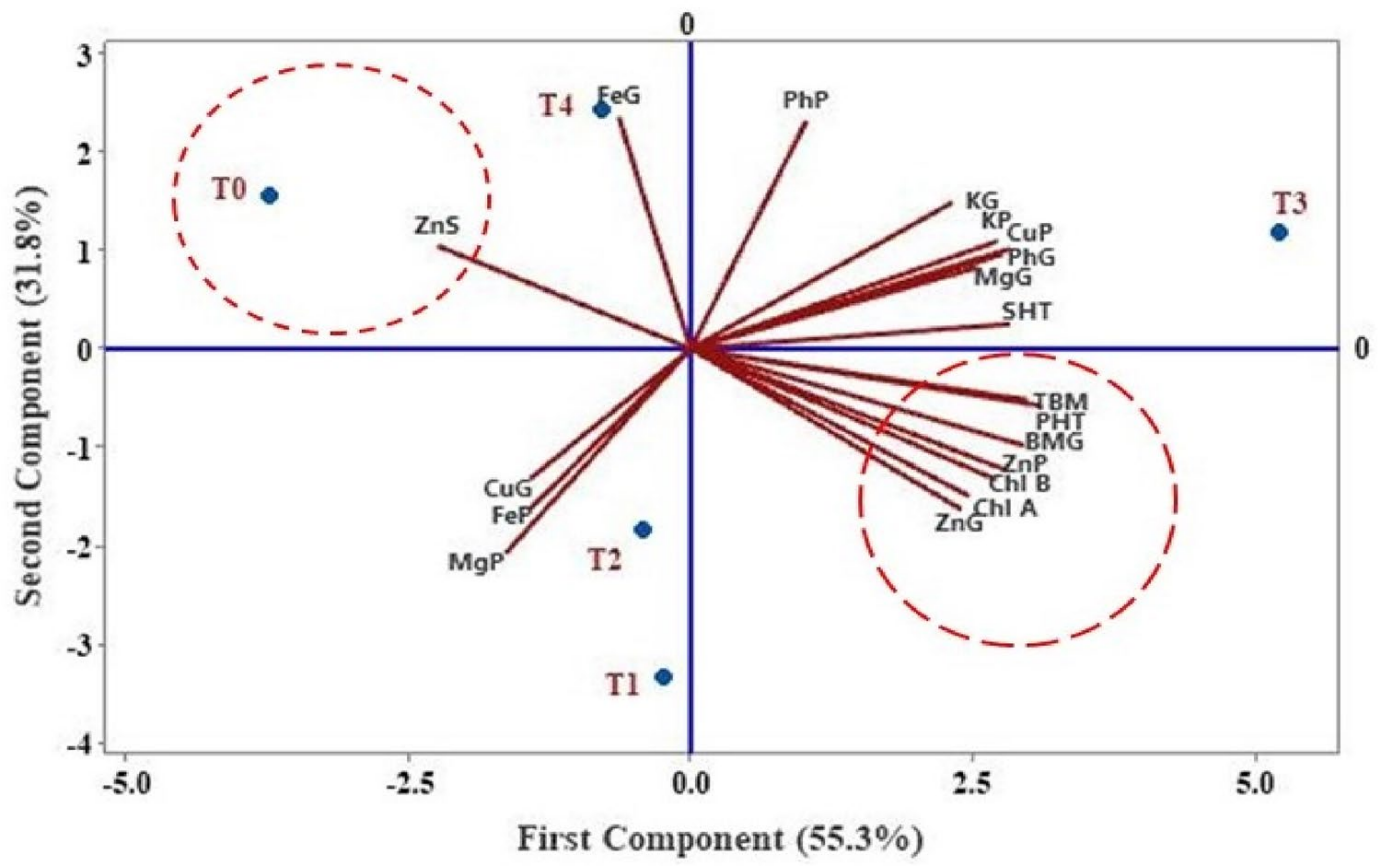
Principal component analysis (PCA) biplot. Zn content in: plant (ZnP), grains (ZnG), soil (ZnS); Mg content in: plant (MgP), grains (MgG); copper content in: plant (CuP), grains (CuG); potassium content in: plant (KP), grains (KG), iron content in: plant (FeP), grains (FeG); phosphorus content in: plant (PhP), grains (PhG); chlorophyll content A & B (Chl A & Chl B); total plant weight (TBM), the total weight of plant grains (BMG), plant height (PHT), spikelet height (SHT).

**Figure 11:**
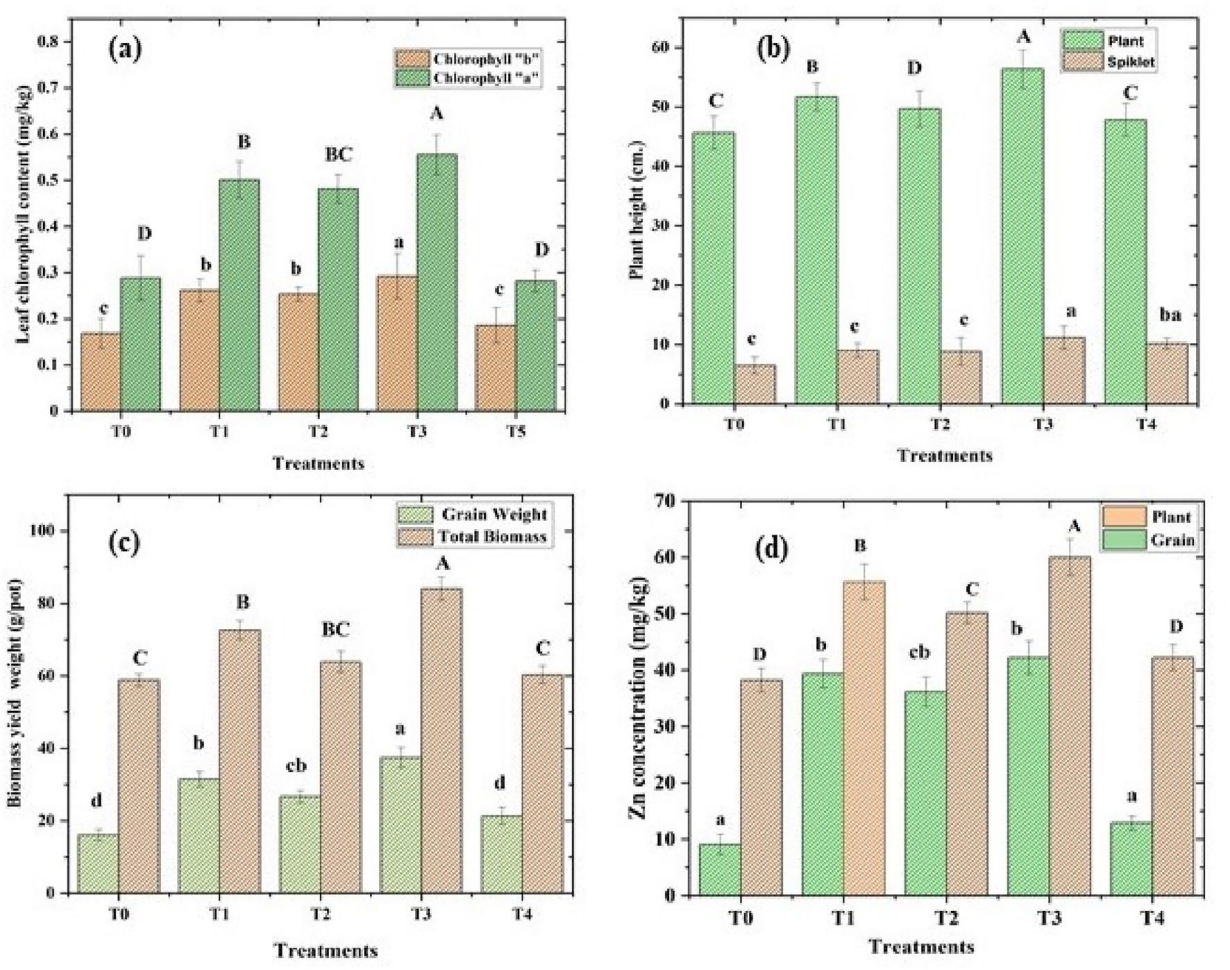
(**a**–**d**) Changes in chlorophyll ‘a &b’ (**a**), the height of the plant and length of the spikelet (b), total biomass yield and weight of grains (**c**), the zinc content in plant body and grain (d); in a pot soil grown wheat treated with various treatments. ( T0 control, T1 ZnSO4.7H_2_O, 75 mg/kg, (T2) ZnO NPs (sheet-like structure) from ZnSO_4_.7H_2_O with one dimension ~17 nm, T3 ZnO NPs (sheet-like structure) with PM 1.4%w/w and (T4) control PM).

**Figure 12:**
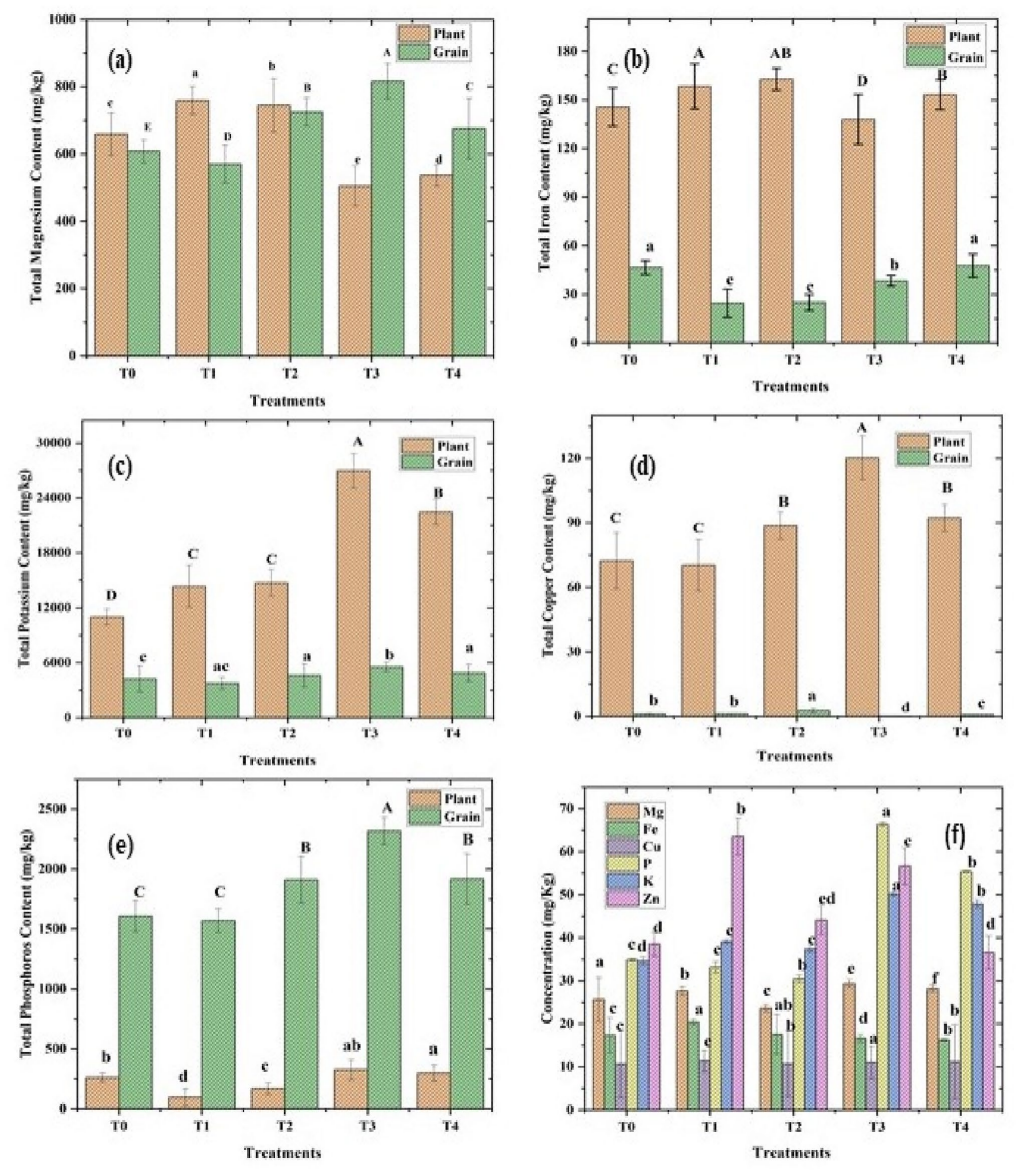
(**a**–**f**) Changes (plant & grain) in total Magnesium (Mg) content (**a**), Total Iron (Fe) content (**b**), total Potassium (K) content (**c**), total copper (Cu) content (d) total Phosphorous (P) content (**e**) and changes in mineral concentration (Mg, Fe, Cu, K, and P) of soil at their natural level (without external input) in soil grown wheat treated with (T0 control, T1 ZnSO4.7H_2_O 75 mg/kg, (T2) ZnO NPs (sheet-like structure) from ZnSO_4_.2H_2_O with one dimension ~17 nm, T3 ZnO NPs (sheet-like structure) with PM 1.4%w/w and (T4) control PM).

**Figure 13:**
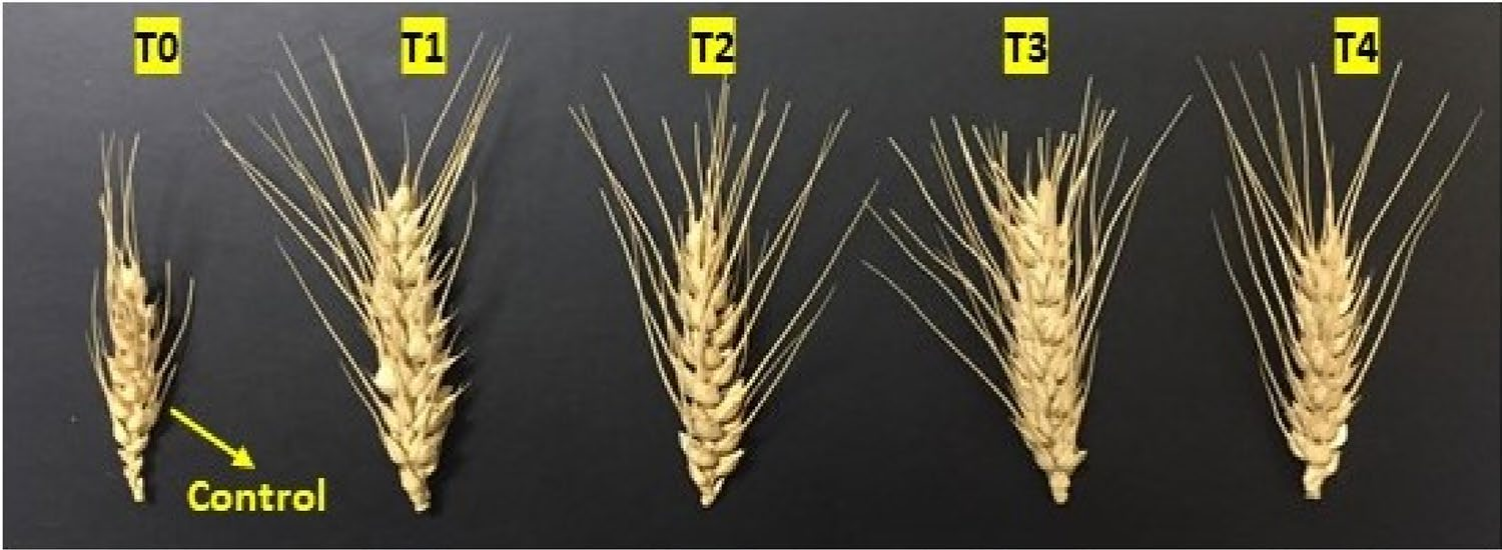
Shows the overall health and the spikelet’s length for the various wheat treatments. (The spikelet has been chosen as an average of the group representing the mean of the corresponding data.

The randomised controlled pot trial with positively charged (+ 7.61mv) ZnO NPs (wurtzite, P63*mc,* 75 mg/kg), 1.4% w/w sugar press mud (PM) and inorganic salt ZnSO_4_.7H_2_O (75 mg/kg) on wheat crop as described in detail in Table 1 and Fig. 9 demonstrated some general trend. The agronomic parameters, like chlorophyll content, plant height, spikelet length, biomass, and grain yield, followed a similar trend of T3 > T2 > T1 > T4 > T0 at a statistically significant difference level (*P* < 0.05) ANOVA analysis. A similar trend was observed for nutritional parameters like *Zn*-fortification in plants and grain. Interestingly, many parameters like soil organic carbon (SOC), biomass yield, plant, and grain metals (Zn, Fe, Cu, P and K) contents in soil–plant continuum were found to be in significant correlation, as shown in Fig. 10. Noticeably, the soil organic carbon (SOC) appears directly correlated with plant metal content, which in turn, was strongly correlated with the agronomic performance of the wheat crop. The correlation between (SOC) and plant metal content could be on account of the instrumental role played by the increased soil organic carbon level due to the addition of sugar dry press mud (1.4 w/w) as shown in the results of T3 treatment compared to others (Fig. S3). The correlation between plant metal content and agronomic performance led by plant *Zn* content is obvious due to the physiological role played by these essential elements in plant metabolism (Fig. 11 and 12). Nearly ~ 42% increase in plant chlorophyll content in T3 treatment followed by the corresponding increase in agronomic performance, i.e. increase in plant height (~ 19%), spikelet length (~ 32%), biomass yield (~ 17%) and grain yield (~ 24%) is evidence by the fact of the increase of plant metal content, especially the zinc (Zn) in the T3 samples (Fig. 11, 12 and 13). This is further supported by the fact of the proportionate removal of nutrients from the soil, as shown in Fig. S2. This difference in T3 treatment with respect to control (T0), (T1), (T2), and (T4) treatment is reasonably due to the mineral mobilising role played by the increased soil colloidal carbon led by PM or its very pH moderating properties. This rationale appears applicable considering the various previous reports highlighting the role of soil organic carbon (SOC) and soil pH in nutrient mobilisation and crop productivity^15,30–34^. However, none of these reports have examined the mechanistic aspects of soil organic carbon-induced nutrient mobilisation in the soil plant continuum^35^. As a preliminary investigation, we have shown that the addition of PM in soil introduces some negatively charged (− 0.27) soil colloidal particles (0.484 μm) in soil solution, which may modify the mineral-soil organic carbon interface or can alter the exchange capacity of the soil. This can also affect the microbial flora or the whole soil mineral ecology, affecting ultimately the nutrient mobilisation^30^. This appears quite feasible according to the different results of the treatments. Moreover, it is hard to say anything about the efficiency of ZnO NPs (50% Zn) against inorganic zinc sulphate salt (22.62% Zn) fertiliser (T2) due to the availability of low zinc content at the same dose of ZnO NPs (75 mg/Kg). However, the differences in the results of T3 treatment with respect to T0, T1 and T4 are certainly due to the role played by the PM. Nevertheless, the varying mass percentage (%) distribution of the metals in the soil–plant continuum is also affected due to the genetic and agronomic conditions of the system^36^. But, under the given genetic and agronomic conditions, the higher concentration of the Zn metal, among others, could be mainly due to the modified *ZnO-SOC* interface^33^. This underlines the value of carbon sequestration into the soil through agro-industrial waste (PM) in maintaining sustainable and efficient crop nutrition in a climate-resilient manner^3^. This appears further encouraging in light of the similar repots highlighting soil organic carbon's role in nutrient mobilisation and environmental sustainability^16,31,33^. However, amid increasing concerns over the bio and environmental compatibility of the poorly defined nanomaterials and lack of enough information over their mechanism and efficiency, this needs vigorous investigation^37,38^.

### Mineral interactions and fortification in plant and soil

Beyond the general effects of PM in making a difference to nutrient mobilisation and crop productivity. Sugar press mud (PM) appears to influence some of the important nutrient interactions in the soil–plant continuum. For instance, Zn-P interactions in soil often leads to decreased uptake of Zn from soil to plant due to the immobilisation (insoluble complex Zn_3_(PO4)_2_ formation) of Zn^2+^ in the presence of phosphate (PO_4_^3-^) anions or vice versa^39^. In this experiment, we observed no significant decrease in phosphorous (P) content in plant samples in zinc treatments rather we found little higher zinc (Zn) content in T3 than T4, whereas lower zinc content in T1 than T2 with respect to control T0 (Fig. 11 and 12). This indicates negative Zn-P interaction in the case of (T1 and T2) whereas a positive interaction in the case of T3 and T4, marking the role of press mud (Fig. 11). This reversal in the nature of interaction can either be due to the pH moderating effect of PM making precipitation of Zn difficult in soil solution or due to the altered adsorption or complexation of zinc in the presence of PM induced colloidal carbon^31,33^. However, this observation and the exact cause need to be further investigated. Moreover, several reports highlight the negative correlation of zinc (Zn) with inorganic phosphate over varied experimental conditions^39^. However, this observed interaction is at the natural level (~ 349 mg/kg) of phosphorous (P) and at the given level of zinc (~ 143 mg/kg) in the soil as no inputs other than ZnO NPs, inorganic Zn salt and PM were added to the treatments. It is worth mentioning here that these nutrient elements (Zn, Fe, Cu, Mg) under study are present in PM (Fig. 2 and Table 2) only in the trace amount (< 0.3%) thus have been ignored. The wheat plant and grain concentration of Zn remain in the range of (35–60 and 10–40 mg/kg) respectively (Fig. 11d). However, the other metal–metal (Zn with Fe, Mg, Cu and K) and metal non-metal (Zn with N) interactions in this study have not shown any striking change in their behaviour pattern due to the intervention of PM, except the extent of the mobilisation of these nutrients. For instance, Zn interaction with N is reported to be synergetic (positive) in various studies, so it has been observed in this case^40,41^. Considering the contrary reports by Ali et al.^42^ and Fageria^43^, the case of redox-active metal iron (Fe) the interaction appears to be complex. In addition, here in our experiment, neither the order of plant and grain iron (Fe) average concentration, i.e. 145.58 to 162.72 mg/kg (T_2_ > T_1_ > T_4_ > T_0_ > T_3_), and 24.51 to 47.72 mg/kg (T_4_ ~ T_0_ > T_3_ > T_2_ ~ T_0_) respectively as shown in (Fig. 12), nor the effect of SOC seems to be related. This makes it difficult to infer anything from this observation. However, though not very clear yet, iron (Fe) appeared to be negatively correlated with soil zinc, probably affecting the Fe uptake, and thus indicating the non-root interface interaction of two metals as suggested by the fact that Zn interferes with Fe transfer from root to shoot^44^. Nonetheless, a detailed investigation needs to be undertaken to unravel the complex interaction of Zn-Fe in the plant continuum. Similarly, Mg and Cu have shown negative and positive correlation with PM in soil and plant, probably due to the two metals have opposite pH preference for bioavailability. Since PM has some pH moderating effect on soil Mg have shown a negative correlation (Fig. 12) with PM treatment (T3 and T4). This is supported by the fact that a study by Prasad et al.^45^ and Fageria^43^ have shown a positive correlation in the absence of any soil amendment. However, copper and zinc, being divalent cation, can compete for binding sites in soil-root interface. Still, such competitions are not predominant because soil Cu concentration often lie in the range of 5–60 mg/kg, which is very small, against the Zn concentration, which is in the 10–300 mg/kg^45^.

Moreover, the mono-positive potassium (K) though, chemically different from Zn, have a strong physiological relationship with zinc, as the deficiency of Zn leads to the exudation of K^+^, amino acids and phenolics in cotton (*Gossypium hirsutum L.*) wheat (*Triticum aestivum L.)* and many other plants grown in solution culture under controlled environment. This is due to the role of zinc in maintaining the integrity of the plasma membrane^45^. For instance, Fageria^43^ has shown that zinc utilisation increases with an increasing rate of K application in corn. However, in this study K has shown strong correlation with PM, probably due to soil's modified cation exchange capacity due to the addition of PM^46^. Thus, the mineral interaction in this study remains largely the same as reported in the literature except the extent of mobilisation and Zn-P interaction. However, the complex behaviour of highly redox active metals i.e. Fe and Cu, should be investigated in greater detail regarding their role in biogeochemical cycle and soil organic matter stabilisation.

## Principal component analysis (PCA)

Principal component analysis (PCA) was undertaken to determine the correlation patterns of variable sets in different soil treatments. PCA biplot (Fig. 10) showing locations of varying soil treatments and correlations among contents of Zn (grain, plant, soil), grain weight, total weight of the plant, height of plant and spikelet, minerals (Mg, K, P, Fe, and Cu) in plants and grains and chlorophyll (a & b) content in different soil treatments with a total variance of 87.1% distributed in principle components 1 and 2 as 55.3% and 31.8% respectively. It is evident from the biplot that there are significant differences among quality variables due to different soil treatments. Contents of Zn in grain and plant, chlorophyll (a & b), the weight of plants and grains, and plant height are in proximity and are showing a positive correlation, while it is interesting to note that these sets of variables are negatively correlated with soil Zn content which explains the commensurate uptake of zinc from soil and its distribution in the plant. Further, proximity can be seen in potassium content in plants and grains, copper content in plants, and phosphorus and magnesium content in the positive loading quadrant. Copper and magnesium contents in plants and grains are negatively correlated with each other due to the translocation of these minerals in the plants.

## Conclusion

In summary, the sheet-like ZnO NPs (~ 17 nm, + 7.61 mv) prepared using sugar press mud (PM) extract when applied (75 mg/kg) to wheat crop through (1.4%w/w) PM amended soil matrix have shown a marked effect on various agronomic as well as nutritional parameters. The treatment having 1.4%w/w sugar press mud and 75 mg/kg of synthesised ZnO nanoparticles has shown ~ 19% zinc (Zn) grain fortification and ~ 24% of yield improvement against the control at a significance level *P* < 0.05 (ANOVA) in a randomised control pot trail having moderately alkaline (pH ~ 8.0) and low Zn soil. This treatment has also shown a marked effect of PM on mobilisation of other minerals (Fe, Cu, Mg, K, and P). A vital interaction (Zn–P) in a soil plant continuum seems to have been reversed due to the interventional of (PM) induced soil colloidal carbon (− 27.9 mv and 0.4864 um). The apparent difference in this treatment (T3) is probably due to this factor. However, these findings are very limited in terms of the apparent complexity of the system, thus inviting further investigations.

### Supplementary Information

Supplementary Information.

## Data Availability

This manuscript includes all the generated or analysed data during the present study (and its Supplementary Information files).
