# Supplementary material for: Green chemistry routed sugar press mud for (2D) ZnO nanostructure fabrication, mineral fortification, and climate-resilient wheat crop productivity
Source: Sci Rep. 2024 Feb 19;14:4074. doi: 10.1038/s41598-024-53682-0 (PMC10876626; doi:10.1038/s41598-024-53682-0)
Supplement: Supplementary file 1 — Supplementary Information. [file 41598_2024_53682_MOESM1_ESM.pdf]

SUPPLEMENTARY INFORMATION

Scientific reports (Springer Nature)

Title: Green chemistry routed sugar press mud for (2D) ZnO nanostructure fabrication, mineral fortification, and climate-resilient wheat crop productivity

Author's name: Lahur Mani Verma[1,4], Ajay Kumar[1], Ashwani Kumar[2], Garima Singh[1], Umesh Singh[1], Shivani Chaudhary[3], Sachin Kumar[3], Anita Raj Sanwaria[1], Pravin P. Ingole[4], Satyawati Sharma[1*]

Fig. S1 Randomized control block design (RCBD) for the pot trials of the ZnO NPs metal oxides

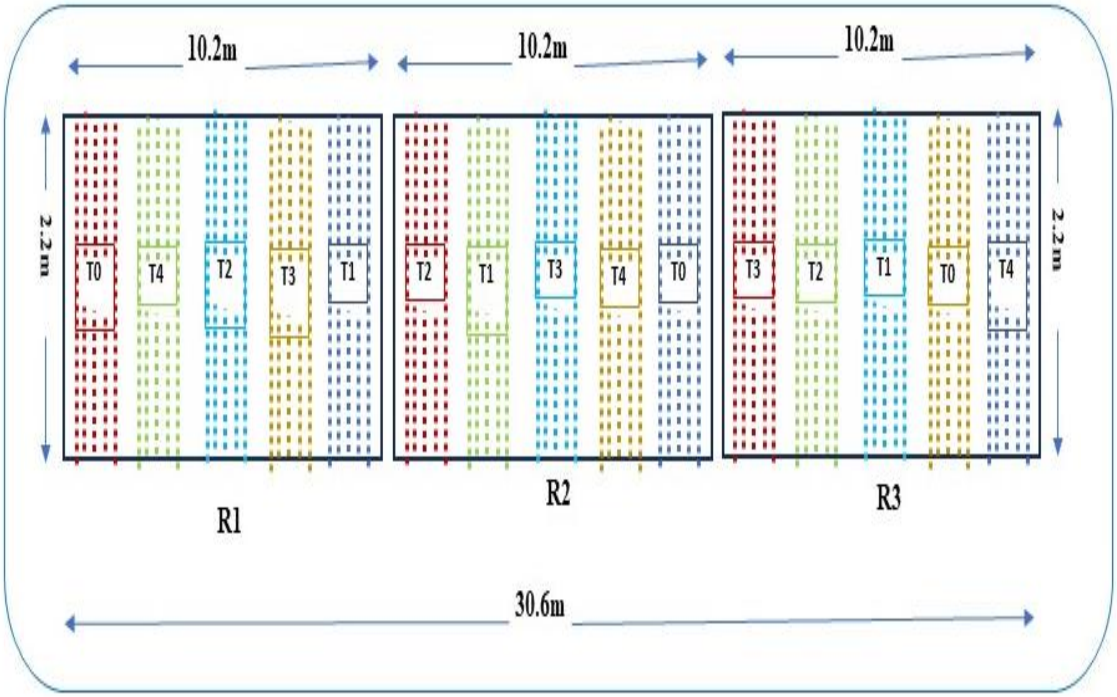

Fig. S2 Soil concentrations of Zn after 90 days of experiments

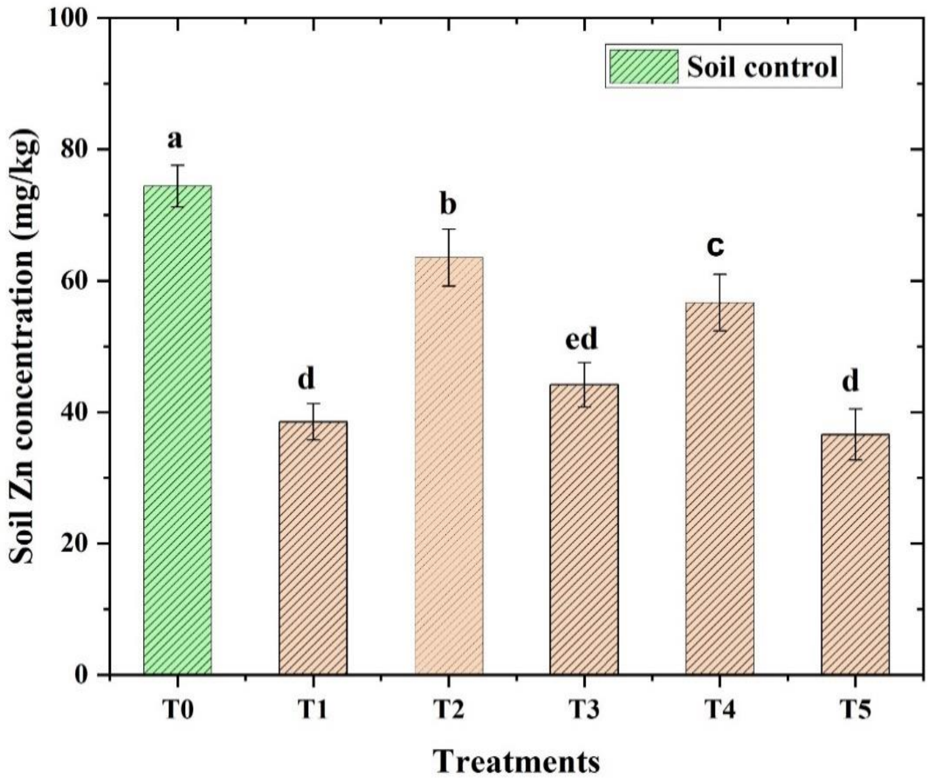

Fig. S3 Correlation of bioavailable soil organic carbon (SOC) with biomass and grain yield.

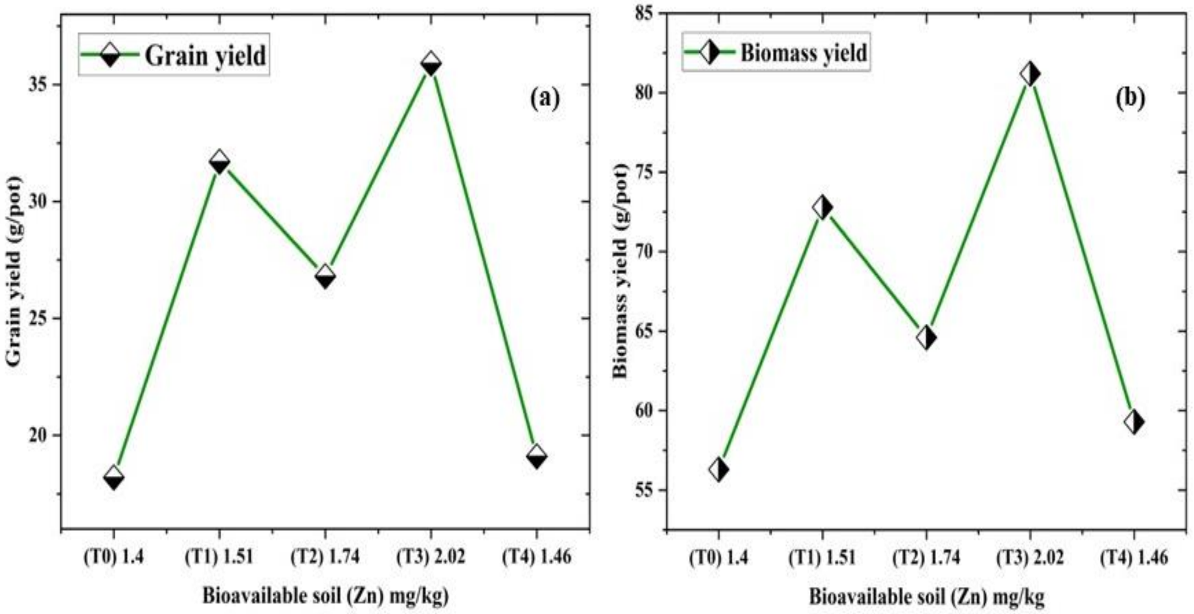

Table S1 Synthesis of ZnO NPs at pH 12.0 and temperature 35.0˚ C.

**Precursor salts**	**Ligand**	**Morphology (shape)**	**Avg. size (nm)**	**Zeta potential (mv)**	**Polydispersity Index (PDI)**	**Yield (w%)**
Zinc Sulphate	PM	sheet-like	17 nm	+7.61	0.325	98.6
Zinc Sulphate	N/A	sphere-like	26 nm	-2.27	0.533	97.9

Table S2 Elemental composition of PM (ICP-MS) analysis

**Elements**	**K**	**Mg**	**P**	**Fe**	**Cu**	**Zn**
**Concentration (ug/g)**	58624.126	7109.542	7714.958	4330.3	3.24	2.21
**RSD**	213.71	114.32	98.56	109.87	2.78	1.21
